# Novel Chemically Modified Curcumin (CMC) Analogs Exhibit Anti-Melanogenic Activity in Primary Human Melanocytes

**DOI:** 10.3390/ijms22116043

**Published:** 2021-06-03

**Authors:** Shilpi Goenka, Sanford R. Simon

**Affiliations:** 1 Department of Biomedical Engineering, Stony Brook University, Stony Brook, NY 11794-5281, USA; sanford.simon@stonybrook.edu; 2 Department of Biochemistry and Cellular Biology, Stony Brook University, Stony Brook, NY 11794-5215, USA; 3 Department of Pathology, Stony Brook University, Stony Brook, NY 11794, USA

**Keywords:** chemically modified curcumins, HaCaT cells, melanosome uptake, HEMn-DP cells, melanogenesis, tyrosinase, dendricity

## Abstract

Hyperpigmentation is a dermatological condition characterized by the overaccumulation and/or oversecretion of melanin pigment. The efficacy of curcumin as an anti-melanogenic therapeutic has been recognized, but the poor stability and solubility that have limited its use have inspired the synthesis of novel curcumin analogs. We have previously reported on comparisons of the anti-melanogenic activity of four novel chemically modified curcumin (CMC) analogs, CMC2.14, CMC2.5, CMC2.23 and CMC2.24, with that of parent curcumin (PC), using a B16F10 mouse melanoma cell model, and we have investigated mechanisms of inhibition. In the current study, we have extended our findings using normal human melanocytes from a darkly pigmented donor (HEMn-DP) and we have begun to study aspects of melanosome export to human keratinocytes. Our results showed that all the CMCs downregulated the protein levels of melanogenic paracrine mediators, endothelin-1 (ET-1) and adrenomedullin (ADM) in HaCaT cells and suppressed the phagocytosis of FluoSphere beads that are considered to be melanosome mimics. All the three CMCs were similarly potent (except CMC2.14, which was highly cytotoxic) in inhibiting melanin production; furthermore, they suppressed dendricity in HEMn-DP cells. CMC2.24 and CMC2.23 robustly suppressed cellular tyrosinase activity but did not alter tyrosinase protein levels, while CMC2.5 did not suppress tyrosinase activity but significantly downregulated tyrosinase protein levels, indicative of a distinctive mode of action for the two structurally related CMCs. Moreover, HEMn-DP cells treated with CMC2.24 or CMC2.23 partially recovered their suppressed tyrosinase activity after cessation of the treatment. All the three CMCs were nontoxic to human dermal fibroblasts while PC was highly cytotoxic. Our results provide a proof-of-principle for the novel use of the CMCs for skin depigmentation, since at low concentrations, ranging from 5 to 25 µM, the CMCs (CMC2.24, CMC2.23 and CMC2.5) were more potent anti-melanogenic agents than PC and tetrahydrocurcumin (THC), both of which were ineffective at melanogenesis at similar doses, as tested in HEMn-DP cells (with PC being highly toxic in dermal fibroblasts and keratinocytes). Further studies to evaluate the efficacy of CMCs in human skin tissue and in vivo studies are warranted.

## 1. Introduction

Melanocytes reside in the stratum basale layer of the epidermis and participate in the protection of skin from the damaging effects of UV light on DNA of keratinocytes by transferring melanin containing melanosomes from the melanocytes to keratinocytes in a parasolic arrangement around their nucleus [1]. Tyrosinase is the central enzyme which regulates the two-step reaction sequence that leads to the conversion of L-tyrosine to L-dihydroxyphenylalanine (L-DOPA) and subsequent oxidation to dopaquinone, the precursor to the final polymeric product, melanin [2]. Melanin provides UV photoprotection and scavenges free radicals; however, an excessive production of melanin in the skin can lead to hyperpigmentation and is associated with medical skin disorders such as melasma, postinflammatory hyperpigmentation (PIH), lentigo senilis (LS) and seborrheic keratosis (SK), which can cause significant psychosocial burdens. Melanocytes connect to neighboring keratinocytes in the epidermis by dendrites; one melanocyte contacts up to 30–40 keratinocytes to transfer melanin [3]. The process involves the synthesis, packaging, transfer and uptake of melanin by keratinocytes [4], which is ultimately responsible for perceived skin coloration. Ongoing elucidation of the possible mechanisms of melanosome transfer to keratinocytes points to four proposed models: a membrane fusion model, an exocytosis-endocytosis model, a cytophagocytosis model, and a shedding-phagocytosis model as four possible mechanisms [5]; the role of phagocytosis in the export of melanosomes has been established as a key feature of these proposed mechanisms. For the efficient export of melanosomes by melanocytes, both the dendrite length and number of dendrites are critical components, as melanocytes can branch out by increasing their number of dendrites to contact multiple keratinocytes that are in close proximity and can also elongate their dendrites to export pigment to multiple keratinocytes surrounding them in the suprabasal layers of the epidermis. The inhibition of melanosome export is emerging as a novel target when designing inhibitors of excessive pigmentation and a large number of reports [6,7,8,9], including our previous studies [10,11,12], have described compounds which can inhibit dendricity and thus have the potential to suppress export of melanosomes.

Adrenomedullin (ADM) is a vasoactive peptide secreted by keratinocytes [13,14]; its role has been implicated in stimulating melanogenesis and increasing dendricity [15]. Endothelin-1 (ET-1), another vasoactive peptide, synthesized and secreted by keratinocytes in response to UV radiation [16,17], binds to the ET-B receptor on melanocytes and has also been shown to enhance melanocyte dendricity [18] and upregulate melanin synthesis in UVB-induced pigmentation [19,20,21]. Specifically, it was shown that ET-1 signaling is involved in the later phase of UVB-induced human pigmentation [22]. ET-1 has been shown to act both as a mitogen as well as a melanogen in cultures of human melanocytes [23]. ET-1 is also released by dermal microvascular endothelial cells and is associated with hyperpigmentary skin disorders of PIH [24], dermatofibroma, lentigo senilis and SK [25,26,27]. ET-1 has been shown to act via the phosphorylation of the downstream target of protein kinase C (PKC), microphthalmia transcription factor (MITF). MITF is a known transcription factor for the tyrosinase gene; it is activated by cAMP signaling after UV irradiation and is considered the master regulator of melanogenesis [28]. MITF also plays a key role in the regulation of melanocyte survival and differentiation [29]. In addition, MITF has a pivotal role in the regulation of melanosome transport by acting on melanosomal proteins that form a complex in which myosin VA and Rab27A are joined by the effector melanophilin (MLPH) [30].

Commercial skin whiteners, such as kojic acid (KA), hydroquinone (HQ) and arbutin (glycosylated HQ) are known to exhibit serious side effects [31,32], prompting a need for safer and nontoxic alternatives for the treatment of hyperpigmentation disorders of the skin [33]. Curcumin, a polyphenolic bioactive compound, isolated from the rhizomes of turmeric, *Curcuma longa*, is a popular nutraceutical compound with a broad spectrum of pleiotropic activities that is used as an oral supplement in the management of various cutaneous conditions [34] and is reported to inhibit cellular melanogenesis [35,36,37,38,39]. Despite this efficacy, the practical use of curcumin in commercial skin whiteners and clinical pharmaceuticals has been met with limited success due to its poor solubility, stability and bioavailability [40], in addition to the bright yellow coloration that is aesthetically unpleasing for personal care applications. In our earlier study [41], we reported on the capacity of four novel CMC analogs to inhibit tyrosinase enzyme activity in an acellular system as well as melanogenesis in B16F10 mouse melanoma cells; we have further begun to elucidate the mechanisms by which they inhibit melanogenesis. In the current study, we have extended our work and evaluated the anti-melanogenic activities of CMC2.14, CMC2.24, CMC2.23 and CMC2.5 in primary human melanocytes derived from darkly pigmented skin to validate the application of these compounds for treatment of hyperpigmentation disorders. In addition, we have also evaluated the effects of CMCs on steps of melanosome export in the pathway of melanogenesis.

## 2. Results

### 2.1. Effect of Compounds on Keratinocyte Viability

**Figure 1:**
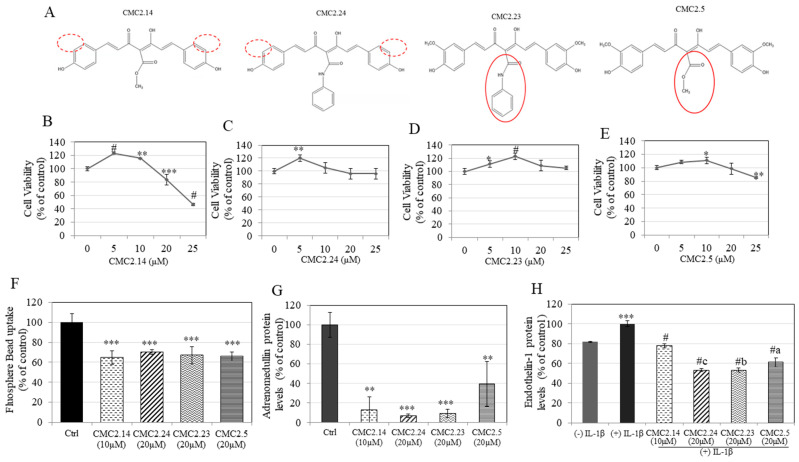
(**A**) chemical structures of CMC2.14, CMC2.24, CMC2.23 and CMC2.5, the red circles show the substituent groups which differ between the CMCs; human keratinocyte (HaCaT) viability in the presence of different concentrations of (**B**) CMC2.14, (**C**) CMC2.24, (**D**) CMC2.23 and (**E**) CMC2.5 measured by MTS assay; (* *p* < 0.05; ** *p* < 0.01; *** *p* < 0.001; # *p* < 0.0001 vs. control. One-way ANOVA with Dunnett’s test); (**F**) inhibition of phagocytosis of FluoSphere beads by HaCaT cells after 24 h exposure to CMC2.14 (10 µM), CMC2.24 (20 µM), CMC2.23 (20 µM) and CMC2.5 (20 µM); (*** *p* < 0.001 vs. control; One-way ANOVA with Dunnett’s post hoc test); (**G**) adrenomedullin protein levels in cultures of HaCaT cells treated with the compounds for 48 h (** *p* < 0.01 and *** *p* < 0.001 vs. Ctrl; One-way ANOVA with Tukey’s post hoc test) and (**H**) endothelin-1 protein levels in cultures of HaCaT cells treated with compounds. (*** *p* < 0.001 vs. (−)IL-1β; # *p* < 0.0001 vs. (+)IL-1β; letter c- *p* < 0.05 vs. CMC2.5; letter b- *p* < 0.05 vs. CMC2.5; letter a- *p* < 0.001 vs. CMC2.14; One-way ANOVA with Tukey’s post hoc test); all data are mean ± SD of triplicates.

In order to assay the four CMCs (chemical structures shown in Figure 1A) for their effects on melanosome export in keratinocytes, we first identified noncytotoxic concentration ranges for all CMCs. Our results showed that CMC2.14 induced significant cytotoxicity at 20 and 25 µM, while it stimulated proliferation of HaCaT cells at lower concentrations of 5 and 10 µM (Figure 1B). CMC2.24 was nontoxic over the range of 5–25 µM and enhanced HaCaT proliferation at 5 µM (Figure 1C). CMC2.23 was nontoxic to HaCaT cells and stimulated proliferation by 11.3% at 10 µM and by 22.9% at 20 µM (Figure 1D). CMC2.5 was significantly toxic to HaCaT cells only at 25 µM, at which point it reduced viability by 15% (Figure 1E). Taken together, the results showed that CMC2.14 was nontoxic over a range of 5–10 µM, and CMC2.5 was nontoxic over a range of 5–20 µM, while the other two CMCs were nontoxic over the broader concentration range of 5–25 µM.

### 2.2. Effect of Compounds on Phagocytosis of FluoSphere Beads by Keratinocytes

Compounds which have the potential to inhibit uptake of melanin by keratinocytes can offer attractive targets for skin pigmentation inhibitors directed to the later stages in the melanogenesis pathway. We selected concentrations for the four CMCs which were nontoxic and potent in their anti-melanogenic activity; the results of the phagocytosis assay are summarized in Figure 1F. All the four CMCs demonstrated similar levels of suppression of bead uptake, which were significant compared to the control. CMC2.14 (10 µM) and CMC2.24 (20 µM) inhibited phagocytosis by 39.6% and 34.4%, respectively. CMC2.23 (20 µM) and CMC2.5 (20 µM) inhibited uptake by 37.4% and 38.4%, respectively.

### 2.3. Effect of Compounds on ADM and ET-1 Protein Levels in Keratinocytes

The levels of ADM protein in supernatants of HaCaT cells were significantly attenuated after treatment with all CMCs (Figure 1G). The mean values of ADM levels for CMC2.14 (tested at 10 µM) were 12.82 ± 13.49% (*p* < 0.001) while for all the other three CMCs (all tested at 20 µM) the ADM levels were 7.01 ± 1.89% (CMC2.24; *p* < 0.001), 9.25 ± 4.42% (CMC2.23; *p* < 0.001) and 39.41 ± 22.83% (CMC2.5; *p* < 0.01). Next, the levels of ET-1, another protein secreted by keratinocytes that can also mediate melanocyte dendricity, were measured in the cultures of keratinocytes stimulated with cytokine IL-1β. IL-1β significantly increased ET-1 secretion (Figure 1H); the levels of ET-1 protein in supernatants of keratinocytes stimulated with IL-1β were significantly downregulated in the presence of all CMCs. CMC2.14 (10 µM) attenuated ET-1 levels by 22.18%, while CMC2.24, CMC2.23 and CMC2.5 (all at 20 µM) attenuated the protein levels by 46.88%, 46.84% and 38.91%, respectively (*p* < 0.001). Additionally, the diminution in ET-1 levels by CMC2.24 and CMC2.23 was statistically greater than that achieved by CMC2.5. 

Taken together, the results demonstrate that all the CMCs possess the capacity to attenuate the levels of ADM and ET-1, two key proteins secreted by keratinocytes that are involved in one or more of the later steps of melanosome export in the pathway of melanogenesis.

### 2.4. Effects of Compounds on Viability of HEMn-DP Cells

**Figure 2:**
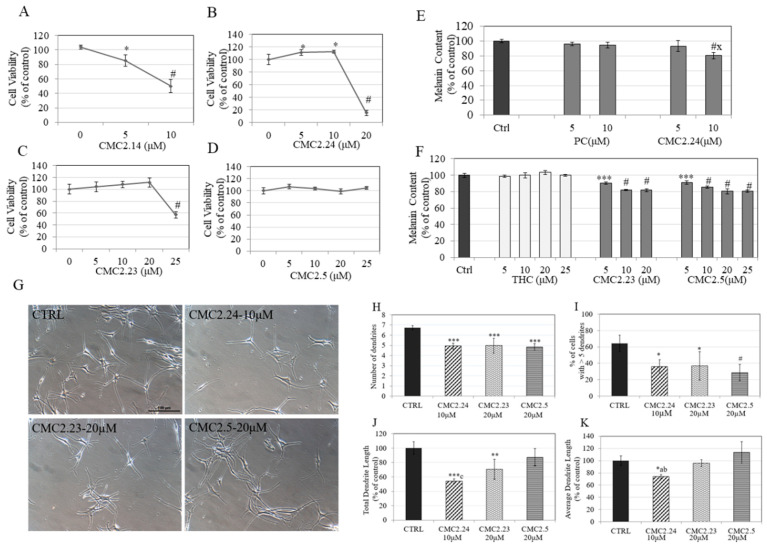
Human primary epidermal melanocyte—darkly pigmented (HEMn-DP) viability in the presence of different concentrations of (**A**) CMC2.14; (**B**) CMC2.24; (**C**) CMC2.23; and (**D**) CMC2.5 for 48 h measured by MTS assay (* *p* < 0.05; # *p* < 0.0001 vs. control. One-way ANOVA with Dunnett’s test). Data are mean ± SD of quadruplicates; melanin content in cultures of HEMn-DP cells treated for 48 h with different concentrations of (**E**) PC and CMC2.24 and (**F**) THC, CMC2.23 and CMC2.5; (*** *p* < 0.001; # *p* < 0.0001 vs. control (Ctrl); letter x- *p* < 0.001 vs. PC-10 µM. One-way ANOVA with Tukey’s test; data are mean ± SD of at least two independent experiments). (**G**) Representative phase-contrast micrographs of HEMn-DP cells treated with 0.16% DMSO (control) and compounds: CMC2.24 (10 µM), CMC2.23 (20 µM) and CMC2.5 (20 µM) at objective magnification 20x; the scale bar shown is 100 microns; quantification of dendricity by (**H**) dendrite number; (**I**) % of cells with >5 dendrites; (**J**) total dendrite length and (**K**) average dendrite length. (* *p* < 0.05; ** *p* < 0.01, *** *p* < 0.001; # *p* < 0.0001 vs. Ctrl; letter c-*p* < 0.01 vs. CMC2.5, letter a-*p* < 0.05 vs. CMC2.23 and letter b-*p* < 0.001 vs. CMC2.23, one-way ANOVA followed by Tukey’s post hoc test); a total of up to 100 cells were counted from each treatment group and the results shown are mean ± SD of values combined from at least two independent experiments.

The CMCs were assayed for any cytotoxicity to HEMn-DP cells before proceeding with experiments on their effects on melanogenesis. Our results showed that CMC2.14 significantly reduced cell viability by 18.66% and 53.86% at concentrations of 5 and 10 µM, respectively (Figure 2A). CMC2.24 apparently induced increased viability at lower concentrations, but was highly cytotoxic at 20 µM, with a significant reduction in viability of 80% (Figure 2B). CMC2.23 induced significant cytotoxicity at the highest concentration of 25 µM (Figure 2C), while CMC2.5 was nontoxic over the full concentration range (Figure 2D). Altogether, CMC2.14 was excluded from further testing of its effects on melanogenic activity due to its high cytotoxicity.

### 2.5. Effects of Compounds on Melanin Synthesis in HEMn-DP Cells

Our results showed that CMC2.24 at 10 µM significantly diminished the melanin levels by 19.66% as compared to control (Figure 2E). CMC2.23 significantly diminished the intracellular melanin levels by 9.38%, 17.68% and 18.10% at concentrations of 5, 10 and 20 µM, respectively, while CMC2.5 significantly diminished the melanin levels by 8.88%, 14.39%, 19.47% and 19.30% at concentrations of 5, 10, 20 and 25 µM, respectively (Figure 2F). Interestingly, both PC over the concentration range 5–10 µM (Figure 2E) and THC over the concentration range 5–25 µM (Figure 2F) were ineffective at diminishing melanin levels in HEMn-DP cells. PC and THC were both tested at concentrations which were found to be nontoxic to these primary human cells (Figure S2).

Altogether, our results revealed that CMCs were more effective at diminution of melanin levels in HEMn-DP cells as compared to the parent compound (PC) as well as the colorless derivative tetrahydrocurcumin (THC), both of which did not have any effect over the low micromolar concentration ranges. Moreover, the levels of diminution in melanin levels (~20%) achieved by CMC2.24 at 10 µM were similar to the levels achieved by CMC2.23 and CMC2.5 at 20 µM.

### 2.6. Effects of Compounds on Dendricity in HEMn-DP Cells

For the effects of CMCs on dendricity, we selected CMC2.24 at 10 µM and the other two CMCs at 20 µM. The qualitative evaluation of photomicrographs of HEMn-DP cells treated with CMCs showed that all CMCs suppressed dendricity as compared to control (Figure 2G). CMC2.24 (10 µM) reduced the dendrite number by 26.43%, while CMC2.23 (20 µM) and CMC2.5 (20 µM) reduced the dendrite number by 25.75% and 27.79%, respectively (Figure 2H). Next, we quantified % of cells with >5 dendrites in the control and CMC-treated groups and the results are summarized in Figure 2I. The mean value of percentage of cells with >5 dendrites in control group (64.41 ± 10.17%) was significantly reduced in the presence of all CMCs. CMC2.24 reduced the percentage of cells with >5 dendrites by 28.19%, CMC2.23 reduced the percentage by 27.56% and CMC2.5 reduced the percentage by 35.70%. The results of total dendrite length (TDL) and average dendrite length (ADL) for all compounds are summarized in Figure 2J,K, respectively. CMC2.24 (10 µM) significantly reduced TDL by 46.09% and ADL by 26% as compared to control. CMC2.23 (20 µM) significantly reduced only TDL by 29.38% but did not affect ADL as compared to control. However, CMC2.5 (20 µM) did not affect either TDL or ADL as compared to control. The mean ADL for cells treated with CMC2.24 (10 µM) was significantly lower than that for cells treated with CMC2.23 (20 µM) and significantly lower than that for cells treated with CMC2.5 (20 µM).

Overall, the potency of the compounds in affecting dendricity followed the order: CMC2.24 (10 µM) > CMC2.23 (20 µM) > CMC2.5 (20 µM).

### 2.7. Effects of Compounds on Intracellular Tyrosinase Activity in HEMn-DP Cells

**Figure 3:**
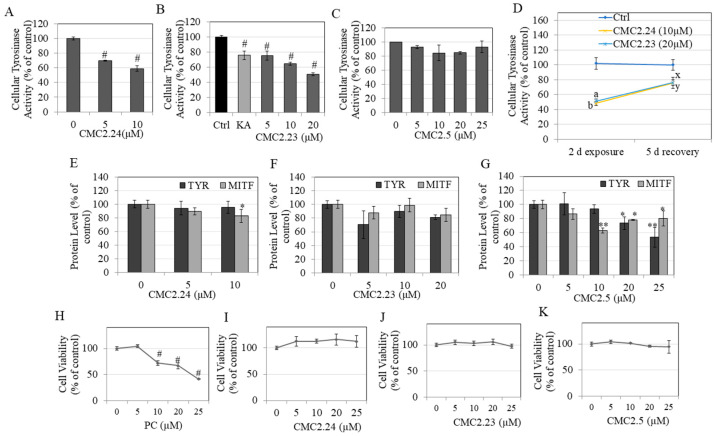
Cellular tyrosinase activity study in HEMn-DP cells treated for 48 h with different concentrations of CMCs; (**A**) CMC2.24; (**B**) CMC2.23; and (**C**) CMC2.5; KA (1 mM) was used as a positive control; (# *p* < 0.0001 vs. control. One-way ANOVA with Dunnett’s test). (**D**) Exposure-recovery study of tyrosinase activity in HEMn-DP cells treated with compounds: CMC2.24 (10 µM) and CMC2.23 (20 µM); letter a- *p* < 0.01 and letter b- *p* < 0.01 vs. control at 2 d exposure; letter x- *p* < 0.05 and letter y- *p* < 0.05 vs. control at 5 d recovery; unpaired *t*-test. Levels of TYR and MITF proteins measured in HEMn-DP cells after treatment with various concentrations of (**E**) CMC2.24; (**F**) CMC2.23; and (**G**) CMC2.5; * *p* < 0.05 and ** *p* < 0.01 vs. control; human dermal fibroblast viability in the presence of different concentrations of (**H**) PC; (**I**) CMC2.24; (**J**) CMC2.23; and (**K**) CMC2.5 for 48 h measured by MTS assay (# *p* < 0.0001 vs. control. One-way ANOVA with Dunnett’s test). Data for (**A**,**B**) are representative of one experiment in triplicates out of two separate experiments. Data for (**C**) are mean ± SD of two separate experiments, while all other data are mean ± SD of triplicates.

Our results showed that CMC2.24 significantly inhibited cellular tyrosinase activity by 30.07% and 40.87% at concentrations of 5 and 10 µM, respectively (Figure 3A), while CMC2.23 significantly inhibited cellular tyrosinase activity by 24.84%, 35.39% and 49.45% at concentrations of 5, 10 and 20 µM, respectively (Figure 3B). On the other hand, CMC2.5 did not have any significant effect on tyrosinase activity at any concentration (Figure 3C). Taken together, our results suggest that the mechanisms of inhibition of melanin production for CMC2.24 and CMC2.23 can be explained, at least in part, by a reduction in the activity of the enzyme tyrosinase. However, the mechanism of action of CMC2.5, which showed levels of suppression of melanin production similar to CMC2.23 at 20 µM, seemed to be unrelated to tyrosinase activity in cells.

### 2.8. Recovery Study of Tyrosinase Activity in HEMn-DP Cells

In order to study the recovery of tyrosinase activity in cells after treatment with CMCs followed by replacement with compound-free medium, we conducted an exposure-recovery study. Our results showed that as expected, CMC2.24 (10 µM) significantly inhibited the tyrosinase activity in HEMn-DP cells by 52.77% while CMC2.23 (20 µM) significantly inhibited the tyrosinase activity by 49.76% after 2 d exposure (Figure 3D). Upon further continuation of HEMn-DP cultures in fresh medium without the compounds for a period of 5 d, the tyrosinase activity in the cells began to recover; cells previously treated with CMC2.24 recovered 28.45% of their tyrosinase activity and cells previously treated with CMC2.24 recovered 25.79% of their activity after culture in compound-free medium (Figure 3D). The tyrosinase activity after the 5 d recovery was still significantly lower than the recovery control group for both compounds; we did not extend the recovery period to see if greater recovery could be achieved.

### 2.9. Tyrosinase and MITF Protein Levels in HEMn-DP Cells

CMC2.24 significantly reduced MITF levels at a concentration of 10 µM but did not affect tyrosinase (TYR) protein level at any concentration (Figure 3E). CMC2.23 at 20 µM showed a trend for reduced TYR and MITF but the levels did not reach statistical significance (Figure 3F). On the other hand, CMC2.5 showed a dose-dependent reduction for TYR protein levels with significant and robust reduction by 26.46% at 20 µM and 46.54% at 25 µM. MITF protein levels were also significantly reduced by 36.95% at 10 µM, 21.83% at 20 µM and 19.83% at 25 µM (Figure 3G).

Overall, the data indicate that CMC2.5 significantly downregulated the levels of both TYR and MITF proteins, while CMC2.24 only inhibited MITF levels. CMC2.23 showed a trend for reduction in protein levels of both TYR and MITF; however, the reductions were not significant. 

### 2.10. Effects of Compounds on Viability of Human Dermal Fibroblasts

The CMCs were also assayed for any cytotoxicity to normal human dermal fibroblasts that are known to have regulatory effects on melanocytes; hence, establishing the nontoxicity of CMCs on fibroblasts would be beneficial. We also evaluated the cytotoxicity of PC to fibroblasts for comparison; our results showed that PC induced significant cytotoxicity to these cells with residual viabilities of 71.93%, 66.92% and 41.17% of the untreated controls after exposure to 10, 20 and 25 µM, respectively (Figure 3H). Our results further showed that CMC2.24 (Figure 3I), CMC2.23 (Figure 3J) and CMC2.5 (Figure 3K) did not exhibit any cytotoxicity over the concentration range tested, indicating the advantage of these analogs as compared to PC.

## 3. Discussion

**Figure 4:**
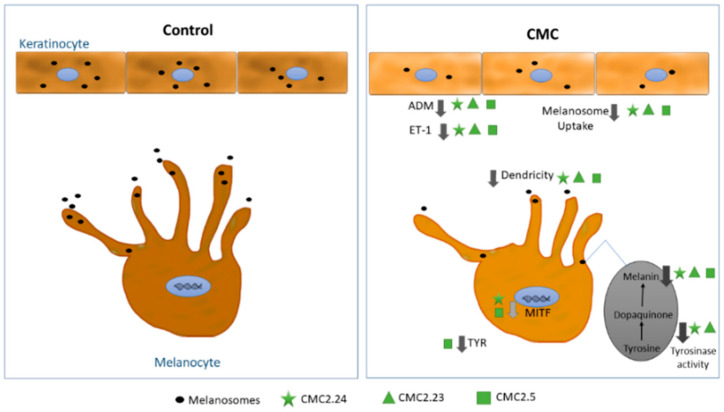
Schematic showing the pleotropic effects by which CMCs inhibit melanogenesis.

Our results demonstrate that of the four CMCs (CMC2.14, CMC2.24, CMC2.23 and CMC2.5) evaluated in this study, CMC2.14 effectively inhibited melanogenesis at the step of melanosome uptake by keratinocytes, but was highly cytotoxic to melanocytes, thus rendering it of no practical use for inhibiting melanogenesis in vivo. On the other hand, the remaining CMCs: CMC2.24, CMC2.23 and CMC2.5 were effective melanogenesis inhibitors and interfered at multiple steps in the melanogenesis pathway by pleotropic modes of action in the absence of cytotoxicity, not only to melanocytes and keratinocytes, but also to dermal fibroblasts. Notably, dermal fibroblasts participate in the cross-talk between keratinocytes and melanocytes and have been shown to be involved in the regulation of paracrine factors in melanogenesis [42,43]. It is noteworthy that the CMCs at low micromolar concentrations showed better efficacy than PC or the colorless hydrogenated derivative, THC (95% tetrahydrocurcuminoids, Sabiwhite™, Sabinsa Corp., East Windsor, NJ, USA) that were used as reference compounds for comparison with CMCs. Moreover, in contrast to the CMCs, PC induced significant cytotoxicity to HaCaT cells, a model human keratinocyte line, at concentrations >10 µM (Figure S1). Since mixtures of compounds have been used in formulations to achieve multifunctional benefits, our primary goal was to test the CMCs and identify the cytotoxicity, anti-melanogenic activity and mechanism of action for the four CMCs separately, in part due to commercial interest in the curcumin derivatives. Both CMC2.24 and CMC2.23 showed similar potency at inhibiting melanin production in human melanocytes and as CMC2.24 has already been reported to exhibit better stability and solubility than the parent compound curcumin, we anticipated that CMC2.23, a close structural analog of CMC2.24, might also demonstrate a similar activity profile. CMC2.5, on the other hand, is another potent candidate which might be effective as a skin-lightening agent, working through a distinct mechanism of action (suppressing tyrosinase protein levels without affecting tyrosinase activity) in addition to its significant antioxidant activity that was reported in our previous study [41]. The various distinct steps at which these CMCs act in melanogenesis have been summarized in a schematic (Figure 4).

We have also evaluated the efficacy of PC and CMCs in moderately pigmented human melanocytes (HEMn-MP) that are derived from Asian skin phototype. We first evaluated any cytotoxicity of these compounds to HEMn-MP cells over a 48 h duration. Our results showed that PC was nontoxic to HEMn-MP cells over a concentration range 5–10 µM (Figure S1A). CMC2.24 was also nontoxic to HEMn-MP cells over a higher concentration range (in contrast to HEMn-DP cells), with significant cytotoxicity at 25 µM (Figure S1B). Lastly, CMC2.23 (Figure S1C) and CMC2.5 (Figure S1D) were nontoxic over the concentration range of 5–25 µM. Our results on intracellular tyrosinase activity showed that PC was less potent (Figure S3A) than CMC2.24 (Figure S3F) at inhibiting tyrosinase activity while CMC2.23 (Figure S3C) was less potent than CMC2.24 but more potent than CMC2.5 (Figure S3H), which did not suppress tyrosinase activity at all. Interestingly, the compounds possess a similar inhibitory profile as that of HEMn-DP cells on tyrosinase activity. The capacity to suppress intracellular melanin production in HEMn-MP cells was also evaluated and indicated that although CMC2.5 was ineffective, both CMC2.24 and CMC2.23 retained their capacity to inhibit melanin production in HEMn-MP cells (data not shown), which suggests that these CMCs are also suitable for diminishing pigmentation in moderately pigmented skin.

Recovery of tyrosinase activity is an important prerequisite for establishing safety, especially since some of the well-known skin whiteners such as HQ and KA cause an irreversible inhibition of tyrosinase activity [44]. A previous study documented the recovery of inhibited tyrosinase activity by novel deoxyarbutin analogs in HEMn-DP cells, establishing their safety [45]. Currently, there are no reports on the recovery by cells after tyrosinase inhibition in cells treated with curcumin or its derivatives, except in our previous study where we evaluated the recovery capacity of B16F10 mouse melanoma cells after treatment with PC and CMCs [41]. To the best of our knowledge, this is the first study to evaluate the recovery of tyrosinase activity by HEMn-DP cells treated with curcumin analogs. Encouragingly, our results of partial recovery of tyrosinase activity of HEMn-DP cells treated with CMC2.24 and CMC2.23 in this study are in agreement with our results in our previous study [41].

Our results present a novel finding that all the CMCs affect not only the synthesis of melanin but may also affect its uptake by keratinocytes as suggested by the results of phagocytosis assay. As it has been shown that keratinocytes do not possess specificity for melanosomes and can phagocytose latex beads in a similar manner to that of melanosomes [46], our use of these beads as a model to mimic melanosome uptake is justified. In addition, we selected a bead size of 0.5 µm that is closest to melanosome size and has been previously used to study the effect of compounds on phagocytosis [47].

A previous study documented that curcumin inhibited melanin production in human melanocytes, in part, by downregulating the levels of MITF protein after a 48 h treatment [48]. Our results on MITF protein levels in HEMn-DP cells treated with CMCs for 48 h showed a significant downregulation of protein levels after exposure to CMC2.5 and CMC2.24, with no significant effect in the case of CMC2.23. MITF has been shown to also regulate melanocyte dendricity [49], hence our results of significant reduction of total dendrite length by CMC2.24 as well as CMC2.5 (although to a lesser extent than CMC2.24) could be explained, at least in part, by the downregulation of MITF protein levels by these compounds.

Dendrite extension and branching is a necessary cellular event needed for the export of melanosomes from melanocytes to keratinocytes. Several compounds have been reported to modulate melanocyte dendricity, which, in part, explains their effects on the subsequent steps of melanogenesis. For example, a combination of natural *Curcuma zedoaria* and aloe vera extracts inhibited melanin synthesis and total dendrite length in mouse melanoma cells [7] and another study reported on another natural extract which inhibited melanin synthesis and total dendrite length in B16F10 cells [6]. Another plant-based compound, methylophiopogonanone B, was shown to induce dendrite retraction and inhibit melanosome transfer in primary human melanocytes [50]. However, the aforementioned reports on natural compounds that suppressed dendricity only reported qualitative results on dendricity without reporting any quantitative data. To date, no study has evaluated the effects of curcumin or its derivatives on melanocyte dendricity. Interestingly, in our previous study, we reported that CMT-308, a second-generation chemically modified tetracycline derivative that shares some structural similarity to CMCs by virtue of its β-diketone group, suppressed dendricity without affecting melanin synthesis [10]. In this study, we evaluated multiple dendricity parameters to elucidate the effects on dendricity by various CMC analogs. Our results indicate that a higher percentage of melanocytes in the control group were polydendritic (>5 dendrites) and that the percentage of polydendritic cells as well as the number of dendrites per cell were reduced significantly after exposure to all of the CMCs; the effects on these two dendricity parameters were similar for all the CMCs we tested. However, the effects of CMCs on total dendrite length and average dendrite length varied after exposure to the three CMCs we studied. The data demonstrate that CMC2.24 exhibited a two-pronged action by suppressing both the dendrite length and dendrite number. However, CMC2.5 only suppressed dendrite number in the absence of changes in dendrite length. Interestingly, our results of a differential response to CMC2.5 in terms of effects on dendricity parameters could be correlated with the results we obtained in keratinocytes, where the levels of ADM, a key protein mediating dendricity, was significantly downregulated after exposure to the CMCs in the order of CMC 2.24 (20 µM) > CMC2.23 (20 µM) > CMC2.5 (20 µM). Taken together, these results demonstrate that CMCs may inhibit melanosome transport by affecting dendrite morphology in a distinctive manner. Dendrite neogenesis does not occur at the same time in all melanocytes and its mechanisms have not been fully separated from dendrite outgrowth [15]. It is not clear if CMCs might attenuate dendrite neogenesis or dendrite outgrowth per se, as those mechanisms have not yet been identified in melanocytes and remain a subject of future investigation. Although we did not further examine whether the capacity of CMCs to inhibit melanosome transfer might be affected in a coculture system where both melanocytes and keratinocytes are in close contact, we believe that the CMCs will possess the capacity to inhibit melanosome export, as they were shown to act on multiple steps of export both in melanocytes and keratinocytes when studied separately. Further studies to test the anti-melanogenic efficacy of CMCs using tissue equivalent models of skin and, we may hope, eventually, in vivo trials are warranted.

Previous studies have documented that endothelin-converting enzyme (ECE-1α), a member of the zinc metalloproteinase family, can catalyze both the conversion of prepro-ET-1, which is synthesized by human keratinocytes, into the inactive precursor Big ET-1 and the subsequent cleavage of Big-ET-1 to yield the mature active form of ET-1 that is then secreted [16,20,51]. Our results of the suppression of ET-1 levels by CMCs may be attributed to the capacity of the CMCs to inhibit the proteolytic processing events associated with ET-1 release, most likely though binding to the zinc atom in the active site of ECE-1α via their β-diketone moiety [52]. In a previous study conducted using an arsenic-induced hyperpigmentation model, the authors reported the involvement of nuclear factor κβ (NF-κβ) in the activation of the ET-1 receptor [53]. CMCs have been previously shown to inhibit NF-κβ activation in an inflammatory periodontitis model [54]. Although we did not evaluate the role of NF-κβ in the suppression of the pro-melanogenic peptides such as ET-1 as a contributor to the anti-melanogenic activity of the CMCs in the current work, future studies to elucidate the mechanistic elements are warranted. The link between vascularization and pigmentation has been reported previously; the activation of VEGF receptor after UVB irradiation has been shown to promote melanogenesis in human melanocytes [55]. A previous study showed that tranexamic acid, a well-known skin whitener, inhibited pigmentation, in part, by suppressing the protein levels of VEGF [56]. Although the anti-angiogenic activity of CMCs has not been sufficiently explored, we have previously shown that CMC2.24 suppressed VEGF secretion in malignant oral cancer cells [57]. Moreover, chemically modified tetracyclines (CMTs), which are structurally similar to CMCs, have shown anti-angiogenic effects on breast cancer cells via the downregulation of VEGF levels [58]. To assess further if the anti-melanogenic capacity of CMC2.24 might also involve a suppression of VEGF levels in melanocytes will also be interesting for future studies.

## 4. Materials and Methods

### 4.1. Materials

The chemically synthesized curcumin derivatives: CMC2.14, CMC2.24, CMC2.23 and CMC2.5 (all 97% purity) were obtained from ChemMaster Int. (Hauppauge, NY, USA). Curcumin (PC, 99% purity) was procured from Selleck Chemicals (Houston, TX, USA). Tetrahydrocurcumin (THC, 96%, Sabiwhite™, Sabinsa Corp., NJ, USA) sample was obtained from Biocogent LLC (Stony Brook, NY, USA). Kojic acid (KA), L-3,4 dihydroxyphenylalanine (L-DOPA) and trypan blue reagent were purchased from Sigma-Aldrich (St. Louis, MO, USA). Heat-inactivated fetal bovine serum (HI-FBS) was procured from R&D Systems Inc. (MN, USA). Dulbecco’s Modified Eagle’s Medium (DMEM; Gibco), phosphate buffered saline (PBS), TrypLE Express (1×) and bicinchoninic acid (BCA) protein determination kit were all procured from ThermoFisher Scientific (MA, USA). Cell-lysis buffer (Cat #: EA-0001) was procured from Signosis Inc. (Santa Clara, CA, USA). The recombinant cytokine, human interleukin-1 beta (IL-1β) was obtained from Miltenyi Biotech (Auburn, CA, USA). FluoSphere beads (0.5 µm, carboxylate-modified) were procured from Molecular Probes Inc. (Eugene, OR, USA).

### 4.2. Cell Culture

Cells of the human keratinocyte line (HaCaT) were obtained from AddexBio (San Diego, CA, USA) and normal human dermal fibroblasts (NHDF), originally procured from Lonza (Basel, Switzerland) were kindly provided by Biocogent LLC (Stony Brook). Both these cells were cultured in DMEM supplemented with 10% heat-inactivated fetal bovine serum (HI-FBS) and 1% antibiotics (penicillin-streptomycin). Human epidermal melanocytes from darkly pigmented neonatal donor (HEMn-DP) were obtained from Cascade Biologics (Portland, OR, USA) and were cultured in Medium 254 (Cascade Biologics) supplemented with 1% human melanocyte growth supplement (HMGS, Cascade Biologics) and 1% antibiotics (penicillin-streptomycin). All cells were cultured in a humidified incubator with 95% air–5% CO_2_ at 37 °C.

### 4.3. MTS Cytotoxicity Assay

For testing cytotoxicity of compounds to HaCaT cells, 2 × 10^4^ cells/well were seeded in a 96-well plate and after 24 h, the test compounds over the concentration range of 5–25 µM were added to the wells which were then incubated for 48 h. At the end of exposure, the culture medium was aspirated and 100 µL of fresh medium containing 20 µL of MTS reagent was added and the plate was incubated at 37 °C for 2 h. Aliquots were transferred to another 96-well plate and the absorbance was read at 490 nm using a Versamax™ microplate reader and the results were expressed as percentage relative to control.

For screening cytotoxicity of compounds to HEMn-DP cells, 3 × 10^4^ HEMn-DP cells/well were seeded in a 96-well plate and after 24 h, the test compounds were added; the cultures were then maintained for another 48 h. After 48 h, MTS assay was conducted according to the method described above, with a shortened incubation time of 90 min.

For testing cytotoxicity of the compounds to normal human dermal fibroblasts (NHDF), 0.5 × 10^4^ cells were dispensed in each well of a 96-well plate and cultured for 24 h, followed by the addition of test compounds and then cultures were maintained for 48 h. At this point, MTS assay was conducted similarly to the method described above. The results were expressed as percentage relative to control.

### 4.4. Phagocytosis Assay in HaCaT Cells

In order to study if CMCs could affect melanosome uptake, we employed a model incorporating FluoSphere beads (0.5 µm) as a model for melanosomes that has been previously well established to study phagocytosis in keratinocytes [59,60]. HaCaT cells were cultured in 24-well plates (3.5 × 10^4^ cells/well) for 48 h, after which the culture medium was replaced with fresh medium containing CMCs (CMC2.14 at 10 µM, CMC2.5 at 20 µM, CMC2.23 at 20 µM and CMC2.24 at 20 µM) or medium containing 0.16% DMSO alone for 48 h. The culture medium was then aspirated and the wells were washed with PBS. At this point, FluoSphere bead solution (sonicated for 15 min) was diluted in complete medium and added to the wells (180 × 10^7^ beads/well) and further incubated for 24 h. After this, the wells were washed in PBS and any residual extracellular fluorescence was quenched using trypan blue (0.1% in PBS). The fluorescence intensity of phagocytosed beads was measured at excitation/emission of 580/605 nm using the well-scan mode (with an average of 21 points/well) in a fluorescence microplate reader (Spectramax^®^ Gemini™ EM, Molecular Devices, San Jose, CA, USA). Controls without any beads were used as a blank for background subtraction. The results are reported as relative fluorescence units (RFU) values and are expressed as percentage of control.

### 4.5. Estimation of Adrenomedullin (ADM) Protein Levels in HaCaT Cells

HaCaT cells were cultured in 6-well plates and treated with test compounds for 48 h. The culture supernatants were then collected from the wells, centrifuged, and stored at −20 °C until assay of ADM levels, using a sandwich-based ELISA kit (My BioSource, San Diego, CA, USA) according to the manufacturer’s instructions. The absorbance was converted to ADM levels (pg/mL) based on the standard curve and values were reported as percentage of control.

### 4.6. Estimation of Endothelin-1 (ET-1) Protein Levels in HaCaT Cells

HaCaT cells were cultured in 35 mm cell culture dishes and treated with compounds in the presence or absence of 10 ng/mL IL-1β for 48 h. IL-1β is a proinflammatory cytokine that is released in chronically sun-exposed as well as acutely UV-irradiated skin [61,62,63]. At the end of treatment, the culture supernatants were centrifuged and stored at −20 °C. The ET-1 protein levels were measured by a sandwich-based ELISA kit (Enzo Life Sciences, Farmingdale, NY, USA) according to the manufacturer’s instructions. The absorbance was converted to ET-1 levels (pg/mL) based on the standard curve and reported as percentage of untreated control.

### 4.7. Estimation of Melanin Levels in HEMn-DP Cells

HEMn-DP cells (2.2 × 10^5^ cells/well) were plated in 12-well plates and cultured for 48 h. The medium was then replaced by medium containing 0.16% DMSO alone as a control or CMCs, and the cultures were incubated for another 48 h. At the end of treatment, the cells were detached, washed in PBS and 250 µL of 1N NaOH was added and heated at 70 °C to solubilize intracellular melanin. The absorbance of the lysates was read at 475 nm and the protein content was estimated by BCA assay. The melanin levels of groups were normalized to total protein and were reported as the ratio: Abs/µg protein. The normalized values were then expressed as percentage of untreated control.

### 4.8. Quantitation of Dendricity in HEMn-DP Cells

HEMn-DP cells (3.5 × 10^4^) were seeded in 6-well plates and cultured for 48 h, followed by the replacement of culture medium with fresh medium containing either solvent control (0.16% DMSO) or CMCs; the plates were then incubated for an additional 48 h. After this exposure, 8–10 random microscopic fields from each well were imaged using a computer-aided image analyzer NIS Elements 5.0 (Nikon Instruments Inc., Melville, NY, USA) and dendrite lengths were measured from center of cell to the tip; the values were then added to give total dendrite length (TDL). The number of dendrites per cell was counted from the images manually and the average dendrite length (ADL) was calculated as the ratio of TDL to the number of dendrites. Finally, to estimate the proportion of multidendritic cells, we manually counted the number of cells with >5 dendrites, which was reported as % of total number of cells.

### 4.9. Determination of Tyrosinase Activity in HEMn-DP Cells

HEMn-DP cells were seeded in 12-well plates at a density of 1.5 × 10^5^ cells/well. After 48 h, the culture medium was replaced by medium containing the test compounds, and the cultures were maintained for 48 h. At the end of treatments, the cells were detached, washed in PBS, and lysed using cell-lysis buffer. The lysates were clarified by centrifugation and 50 µL aliquots were transferred to a 96-well plate with the addition of 150 µL of 3 mM L-DOPA substrate solution. The progress of the reaction was monitored at 475 nm for a period of 40 min at 30 °C with 30 s interval between reads, using a microplate reader in kinetic mode. The tyrosinase activity was determined from the slope of the linear range of the reaction and was expressed as percentage of control after normalization to the total protein contents.

### 4.10. Recovery of Tyrosinase Activity in HEMn-DP Cells

In order to assess whether inhibition of cellular tyrosinase activity by compounds was irreversible, we determined whether the intracellular tyrosinase activity of HEMn-DP cells that had been exposed to compounds could be restored after further incubation of the cells in compound-free medium. Reversibility of inhibition of activity can be considered to be a parameter reflecting safety of the compounds, as an irreversible inhibition of tyrosinase activity might indicate greater damage to the biosynthetic capacity of the melanocytes. CMCs were used at the highest concentration that gave potent melanin inhibition without cytotoxicity (CMC2.24—10 µM, CMC2.23—20 µM and CMC2.5—20 µM). Briefly, HEMn-DP cells were plated in 6-well plates at 2.3 × 10^5^ cells/well and grown for 3 d before addition of CMCs; the cultures were then maintained for an additional period of 2 d. At this point, one set of cultures (exposure group) was processed for the determination of intracellular tyrosinase activity and another set of cultures was continued with fresh medium in the absence of compounds for a further duration of 5 d (with one medium change in between). At the end of the recovery period, these cultures were processed to determine tyrosinase activity for the recovery group. All data were normalized to total protein content and expressed as percentage of untreated controls.

### 4.11. Estimation of MITF and TYR Protein Levels in HEMn-DP Cells

HEMn-DP cells were cultured in 96-well plates for 48 h followed by the replacement with fresh medium containing the test compounds and then cultures were maintained for another 48 h. MITF and tyrosinase protein levels were assayed using an MITF cell-based ELISA (Lifespan Biosciences Inc., Seattle, WA, USA) and a tyrosinase cell-based ELISA (Lifespan Biosciences), respectively, according to the manufacturer’s instructions. The optical densities from the ELISA readings were normalized to total cell mass in each well, as determined by crystal violet staining; the results were reported as percentage of untreated control.

### 4.12. Statistical Analysis

One-way analysis of variance (ANOVA) with Tukey’s or Dunnett’s post hoc test was used for comparison between multiple groups while unpaired *t*-test with Welch correction was used for comparison between two groups. All the analyses were conducted using GraphPad Prism version 8.0 for Windows (GraphPad Software, San Diego, CA, USA, www.graphpad.com, accessed on 7 May 2021) and the differences were considered statistically significant at *p* < 0.05. All data are reported as Mean ± SD.

## 5. Conclusions

In summary, our results provide a proof-of-principle for the use of CMCs (CMC2.24, CMC2.23 and CMC2.5) for the inhibition of melanin synthesis as well as inhibition of one or more subsequent steps of melanogenesis that involve melanocyte dendricity and melanosome uptake. In addition, our results demonstrate that CMC2.14 was not suitable for use as a melanogenesis inhibitor in darkly pigmented melanocytes due to significant cytotoxicity. Our results showed that CMCs are far superior to PC, which is intensely colored, and its colorless hydrogenated derivative, tetrahydrocurcumin (currently marketed as Sabiwhite™), since at low micromolar ranges tested (5–25 µM), the synthetic CMCs (CMC2.24, CMC2.23 and CMC2.5) were more potent in their anti-melanogenic activity than PC and THC, both of which were ineffective at diminution of melanogenesis when tested at similar doses on primary human melanocytes (with PC being highly toxic to dermal fibroblasts and keratinocytes). Moreover, the color of CMCs at such low doses might not hinder their use in cosmetic formulations. Since earlier studies have established the safety and toxicology of the lead compound CMC2.24, it merits further evaluation to be repurposed for treatment of hyperpigmentation disorders in dermatology or as an adjuvant to depigment melanomas for therapeutic purposes. CMC2.23 and CMC2.5 also hold promise as candidates for use in skin-lightening cosmetic formulations. Further studies on the development of an excipient for delivery of these novel compounds in formulations suitable for skin treatment are currently ongoing.

## 6. Patents

SG and SRS are listed as inventors on the patent application describing the use of curcumin analogs for inhibition of human melanogenesis (U.S. Patent 10,300,000). These patent applications have been fully assigned to their institutions, the Research Foundation of Stony Brook University and to ChemMaster International, Inc.

## Data Availability

Not applicable.

## Supplementary Materials

Supplementary Materials can be found at https://www.mdpi.com/article/10.3390/ijms22116043/s1. Figure S1: Viability of HaCaT cells treated with PC at various concentrations (0–25 µM) over a duration of 48 h measured by MTS assay. (* *p* < 0.05; # *p* < 0.01 vs. control. One-way ANOVA with Dunnett’s test.) Data are mean ± SD of triplicates. Figure S2: Viability of HEMn-DP cells treated with (A) PC and (B) THC at different concentrations for a duration of 48 h measured by MTS assay; data are mean ± SD of triplicates. Figure S3: Viability of human epidermal melanocytes—moderately pigmented (HEMn-MP) cells after treatment with (A) PC; (B) CMC2.24; (C) CMC2.23; and (D) CMC2.5 for 48 h. Tyrosinase activity in lysates of HEMn-MP cells treated with (E) PC; (F) CMC2.24; (G) CMC2.23; and (H) CMC2.5 for 48 h. (** *p* < 0.01 and *** *p* < 0.001 vs. control. One-way ANOVA with Dunnett’s test.) All data are mean ± SD of triplicates.
