# Supplementary material for: Novel Chemically Modified Curcumin (CMC) Analogs Exhibit Anti-Melanogenic Activity in Primary Human Melanocytes
Source: Int J Mol Sci. 2021 Jun 3;22(11):6043. doi: 10.3390/ijms22116043 (PMC8199869; doi:10.3390/ijms22116043)
Supplement: Supplementary file 1 [file ijms-22-06043-s001.zip › ijms-1232843-supplementary.pdf]

SUPPLEMENTARY INFORMATION

Novel chemically-modified curcumin (CMC) derivatives exhibit melanogenesis inhibitory activity in human melanocytes

Shilpi Goenka[1,*], and Sanford R. Simon[1,2,3]

[1]Department of Biomedical Engineering, Stony Brook University, Stony Brook, NY 11794-5281, USA
[2]Department of Biochemistry and Cellular Biology, Stony Brook University, Stony Brook, NY 11794-5215, USA
[3]Department of Pathology, Stony Brook University, Stony Brook, NY 11794, USA

* Correspondence
Shilpi Goenka
Department of Biomedical Engineering
Stony Brook University, Stony Brook, NY 11794-5281.
email: shilpi.goenka@stonybrook.edu

1. *MTS Assay for Moderately-pigmented melanocytes*

For screening cytotoxicity of PC, CMC2.24, CMC2.23 and CMC2.5 to moderately-pigmented (MP) melanocytes, we conducted MTS cytotoxicity assay. Briefly, 3 x 10^4 cells /well were seeded in a 96-well plate and after 24 h, compounds were added to the wells and controls were treated with 0.16% DMSO, and cells were cultured for 48 h. After this, the medium was aspirated, and MTS reagent was added and incubated for 90 minutes and absorbance was read at 490 nm using a microplate reader. The results are expressed as % relative to control.

2. *Cellular Tyrosinase Assay for moderately-pigmented melanocytes*

2.2 × 105 HEMn-MP cells were seeded in each well of a 12-well plate and cultured for 48 h, after which the culture medium was replaced by fresh medium containing the test compounds, and cultures were maintained for another 48 h. At the end of treatments, cells were detached, washed in PBS, and lysed. The lysates were clarified by centrifugation and 50 µL were aliquoted in a 96-well plate with the addition of 150 µL of 3 mM L-DOPA substrate solution. The progress of the reaction was monitored at 475 nm for a period of 40 min at 30oC (with 30 sec interval) in kinetic mode. The tyrosinase activity was determined from the slope of the linear range of the reaction and expressed as % of control.

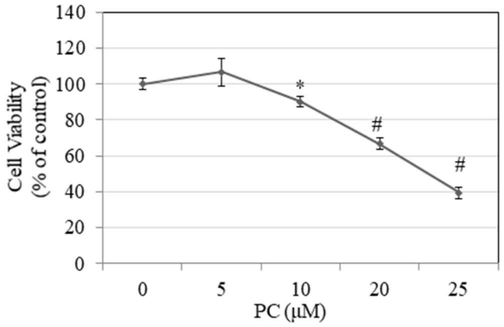

Figure S1: Viability of HaCaT cells treated with PC at various concentrations (0 – 25 μM) over a duration of 48 h measured by MTS assay. (*$p < 0.05$; #$p < 0.01$ vs. control. One-way ANOVA with Dunnett's test); Data is mean ± SD of triplicates.

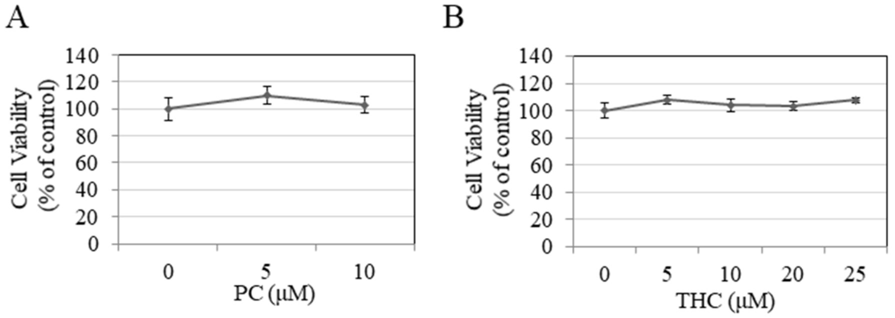

Figure S2: Viability of HEMn-DP cells treated with **A)** PC and **B)** THC at different concentrations for a duration of 48 hours measured by MTS assay. Data is mean ± SD of triplicates.

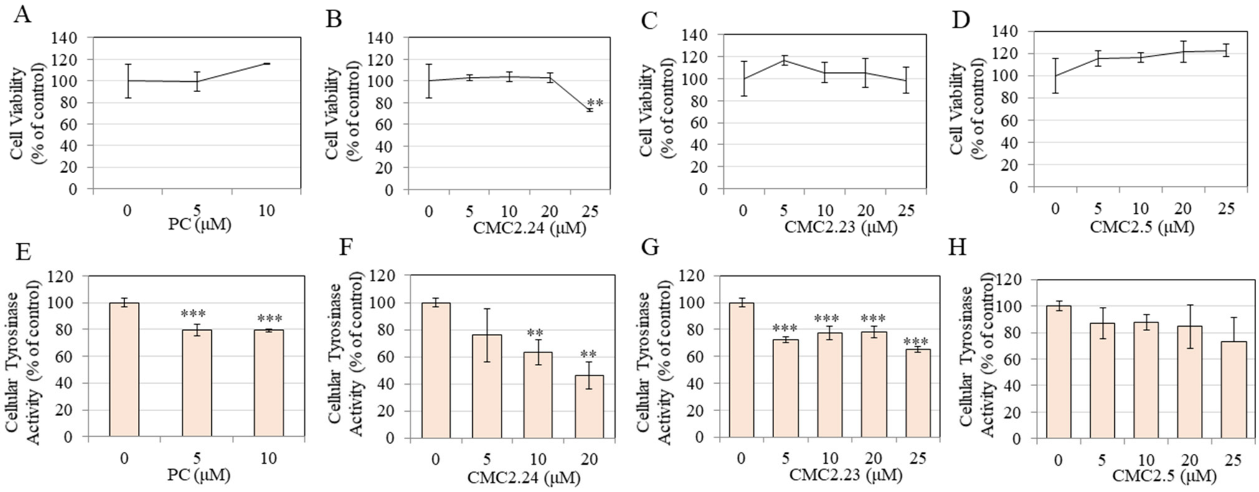

Figure S3: Viability of human epidermal melanocytes-moderately-pigmented (HEMn-MP) cells after treatment with **A)** PC; **B)** CMC2.24; **C)** CMC2.23; and **D)** CMC2.5 for 48 hours. Tyrosinase activity in lysates of HEMn-MP cells treated with **E)** PC; **F)** CMC2.24, **G)** CMC2.23, and **H)** CMC2.5 for 48 hours. (**p < 0.01 and ***p < 0.001 vs. control, One-way ANOVA with Dunnett's test); All data is mean ± SD of triplicates.
